# Comprehensive single‐cell profiling of monocytes in HLA‐B27‐positive ankylosing spondylitis with acute anterior uveitis

**DOI:** 10.1002/mco2.759

**Published:** 2024-10-28

**Authors:** Huan Li, Xueming Ju, Lixin Zhang, Jing Zhu, Jing Zhang, Jialing Xiao, Ting Wang, Weijia Wu, Liang Wang, Chengzi Gan, Xiangmei Li, Yutong Wei, Siyu Zhu, Yu Zhou, Bolin Deng, Ning Xiao, Bo Gong

**Affiliations:** ^1^ Department of Health Management Sichuan Academy of Medical Sciences & Sichuan Provincial People's Hospital University of Electronic Science and Technology of China Chengdu Sichuan China; ^2^ Department of Human Disease Genes Key Laboratory of Sichuan Province and Institute of Laboratory Medicine Sichuan Academy of Medical Sciences & Sichuan Provincial People's Hospital University of Electronic Science and Technology of China Chengdu Sichuan China; ^3^ Department of Research Unit for Blindness Prevention of Chinese Academy of Medical Sciences (2019RU026) Sichuan Academy of Medical Sciences & Sichuan Provincial People's Hospital University of Electronic Science and Technology of China Chengdu Sichuan China; ^4^ Department of Rheumatology and Immunology Sichuan Academy of Medical Sciences & Sichuan Provincial People's Hospital University of Electronic Science and Technology of China Chengdu Sichuan China; ^5^ Department of Ophthalmology Sichuan Academy of Medical Sciences & Sichuan Provincial People's Hospital University of Electronic Science and Technology of China Chengdu Sichuan China

**Keywords:** acute anterior uveitis, ankylosing spondylitis, human leucocyte antigen (HLA)‐B27 positive, monocyte subsets, single‐cell RNA sequencing

## Abstract

Acute anterior uveitis (AAU) is a common extra‐articular manifestation of ankylosing spondylitis (AS), particularly in patients positive for the human leucocyte antigen (HLA)‐B27 genetic marker. To explore the underlying mechanisms of HLA‐B27^+^ AS‐associated AAU, we employed single‐cell RNA sequencing to profile the transcriptomes of peripheral blood mononuclear cells in three HLA‐B27^+^ AS‐associated AAU patients and three healthy controls (HCs). We identified 11 distinct immune cell clusters, with a particular focus on monocytes, revealing six subsets, including three previously unidentified subsets, namely, GTPase immune‐associated proteins, Th17‐related, and lncRNA monocytes, with unique gene expression patterns. Significant differences in monocyte composition, activation states, and gene expression were observed between patients and HCs, particularly within HLA monocyte subpopulations. Notably, enhanced expression of X‐inactive specific transcript and myeloid cell nuclear differentiation antigen genes was validated across monocyte subclusters in patients. Gene Ontology and Kyoto Encyclopedia of Genes and Genomes analysis highlighted significant enrichment in antigen processing and presentation pathways, shedding light on the disease's molecular mechanisms. These findings provide novel insights into the molecular mechanisms of HLA‐B27^+^ AS‐associated AAU and may contribute to the development of targeted diagnostic and therapeutic strategies. Further clinical validation is essential.

## Graphical abstract

Characterization of the immune system in HL‐B27^+^ AS‐associated AAU patients (AAU+AS) and healthy controls (HCs). (A) scRNA‐seq procedure. (B) UMAP plot of 65,712 cells (AAU+AS: 34,492; HCs: 31,220), color‐coded by sample type (left) and cluster (right). (C) Correlation heatmap of the 11 clusters, with red indicating positive and blue indicating negative correlations.

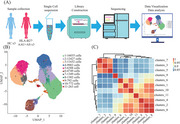

## 1 INTRODUCTION

Autoimmune uveitis (AU) accounts for the intraocular inflammation that may threaten eyesight, and it accounting for approximately 10% of severe visual impairment patients globally and 25% of legal blindness cases among developing countries.^1^, ^2^, ^3^, ^4^ Acute anterior uveitis (AAU) has the highest prevalence in uveitis. Up to 50% of anterior uveitis patients show positiveness for human leucocyte antigen (HLA)‐B27.^5^ As verified in some epidemiological studies, systemic diseases are highly prevalent among AAU cases, mainly including seronegative spondyloarthropathies, like reactive arthritis or ankylosing spondylitis (AS).^6^, ^7^, ^8^ These associations suggest a strong immunogenetic link between AAU and systemic autoimmune diseases, particularly those involving HLA‐B27. AS‐related AAU displays the highest incidence among HLA‐B27‐positive cases, which exhibits certain different clinical features, such as male predominance, early onset age, high fibrinous reaction and hypopyon generation rate, unilateral lesion or alternation between two eyes, anterior predominance compared with posterior uveitis, and many ocular complications.^9^, ^10^, ^11^ However, although its clinical features are well described, the pathogenesis of HLA‐B27^+^ AS‐associated AAU disorder is still unknown. Understanding the specific molecular and cellular mechanisms involved in this condition is crucial for developing targeted therapies and improving patient outcomes. Additionally, reliable disease‐specific biomarkers are lacking at present for the objective and accurate evaluation of in vivo immune status in diverse disease stages or the prediction of therapeutic response.

Much knowledge has been accumulated on the fact that uveitis is greatly driven by dysfunction of T cell‐dependent immunity. Effector CD4+ T (Teff) cells, generally T helper, Th1, and (Th)−17 cells, are important for AU pathogenesis.^12^, ^13^, ^14^ Meanwhile, in some studies, the elevation in Teff cell and/or the reduction in Treg cell counts mediate AU occurrence and development.^15^, ^16^ Recent research has also begun to explore the complex interactions between different T cell subsets and other immune cells, such as monocytes, which could play a pivotal role in disease progression. Based on increasing evidence obtained in human specimens with distinct AU types, IL‐23/IL‐17 signaling pathway is activated in the above disorders.^17^, ^18^, ^19^ However, there is a dearth of studies emphasizing uveitis associated with AS.

Monocytes, tissue‐resident macrophages, B cells and dendritic cells (DCs) represent dominant antigen‐presenting cells, which are closely associated with various inflammatory disorders, tumorigenesis and angiogenesis.^20^, ^21^, ^22^ As it is difficult to obtain eye tissue specimens in uveitis patients (like inflamed retina and uveal tract), the immune pathogenesis is mostly understood by analyzing peripheral blood leukocytes. Monocytes play a critical role in sensing inflammatory environmental alterations, spreading inflammation systemically, and promoting aberrant immune factor synthesis in human autoimmune disorders like rheumatoid arthritis (RA) and multiple sclerosis. Given their central role in mediating immune responses, understanding the behavior of monocyte subpopulations in AAU could provide valuable insights into the disease's underlying mechanisms. Nonetheless, how monocyte populations to uveitis in human beings is still unclear. Consequently, the present work focused on further depicting monocyte subpopulation landscapes and gene expression patterns, so as to understand their pathogenic roles in HLA‐B27^+^ AS‐associated AAU disease activity and treatment response and identify relevant biomarkers.

Single‐cell RNA sequencing (scRNA‐seq) is the effective and unbiased approach to characterize cell types within complicated normal and diseased tissues.^23^, ^24^ This technology allows for the dissection of cellular heterogeneity at an unprecedented resolution, making it particularly useful in complex diseases like AAU. It helps understand disease pathogenesis and is significant for discovery novel therapeutic targets against cardiovascular diseases. For the time being, scRNA‐seq is not applied in characterizing HLA‐B27^+^ AS‐associated AAU. Applying scRNA‐seq in this context could reveal novel cellular subsets and gene expression profiles, providing a more detailed understanding of the disease.

The present work applied scRNA‐seq in mapping immune cell maps of peripheral blood mononuclear cells (PBMC) from HLA‐B27^+^ AS‐associated AAU patients and healthy controls (HCs). We observed that HLA‐B27^+^ AS‐associated AAU showed significant transcriptomic alterations of diverse immune cells within PBMC. Moreover, bioinformatics analysis was conducted for exploring differences in cell clusters and identifying differentially expressed genes (DEGs) probably affecting AAU development, thus providing novel anti‐AAU therapeutic targets.

## 2 RESULTS

### 2.1 Clinical symptoms and laboratory data

This study involved six participants, including three HLA‐B27^+^ AS‐associated AAU patients and three HCs. Table 1 presents the baseline and clinical data of all participants. The HCs were carefully matched with the patient group based on age and sex. Males accounted for 67% of the HLA‐B27^+^ AS‐associated AAU group and 100% of the HC group. The mean ages were 34 ± 4.36 years for the patient group and 36.67 ± 3.79 years for the HC group. All patients were HLA‐B27 positive, with an average disease duration of 1.41 years. Compared with HCs, the HLA‐B27^+^ AS‐associated AAU patients showed significantly higher levels of leukocytes, neutrophils, erythrocyte sedimentation rate (ESR) and C‐reactive protein (CRP) (all *p* < 0.05). The numbers of monocytes, lymphocytes, hemoglobin, and platelets were similar between the two groups (all *p* > 0.05).

**TABLE 1:** General clinical data in the HL‐B27^+^ AS‐associated AAU and HCs groups.

	HL‐B27^+^ AS‐associated AAU group (*n* = 3)	HCs group (*n* = 3)	Reference range	*p* Value
Age (years)	34 ± 4.36	36.67 ± 3.79	NA	>0.05
Sex (male/female)	2/1	3/0	NA	NA
disease duration	1.41	NA	NA	NA
HLA‐B27 positive	3/3	NA	NA	NA
Leukocytes (×10^9^/L)	9.41 ± 1.83	5.32 ± 0.55	4.0–10.0	<0.05
Monocytes (×10^9^/L)	0.53 ± 0.03	0.45 ± 0.06	0.1–0.6	>0.05
Lymphocytes (×10^9^/L)	2.58 ± 1.56	2.21 ± 0.33	0.8–4.0	>0.05
Neutrophils (×10^9^/L)	7.16 ± 1.20	2.57 ± 0.32	2.5–7.5	<0.05
CRP (mg/L)	7.81 ± 6.11	3.40 ± 1.06	5–10	<0.05
ESR (mm/H)	40.67 ± 25.57	12.10 ± 0.49	0–15	<0.05
Hemoglobin (g/L)	118.67 ± 16.65	136.28 ± 8.11	120–160	>0.05
Platelet (×10^9^/L)	261.33 ± 110.82	203.24 ± 16.07	100–300	>0.05

### 2.2 Single‐cell transcription atlas for PBMCs among HLA‐B27^+^ AS‐associated AAU patients

**FIGURE 1:**
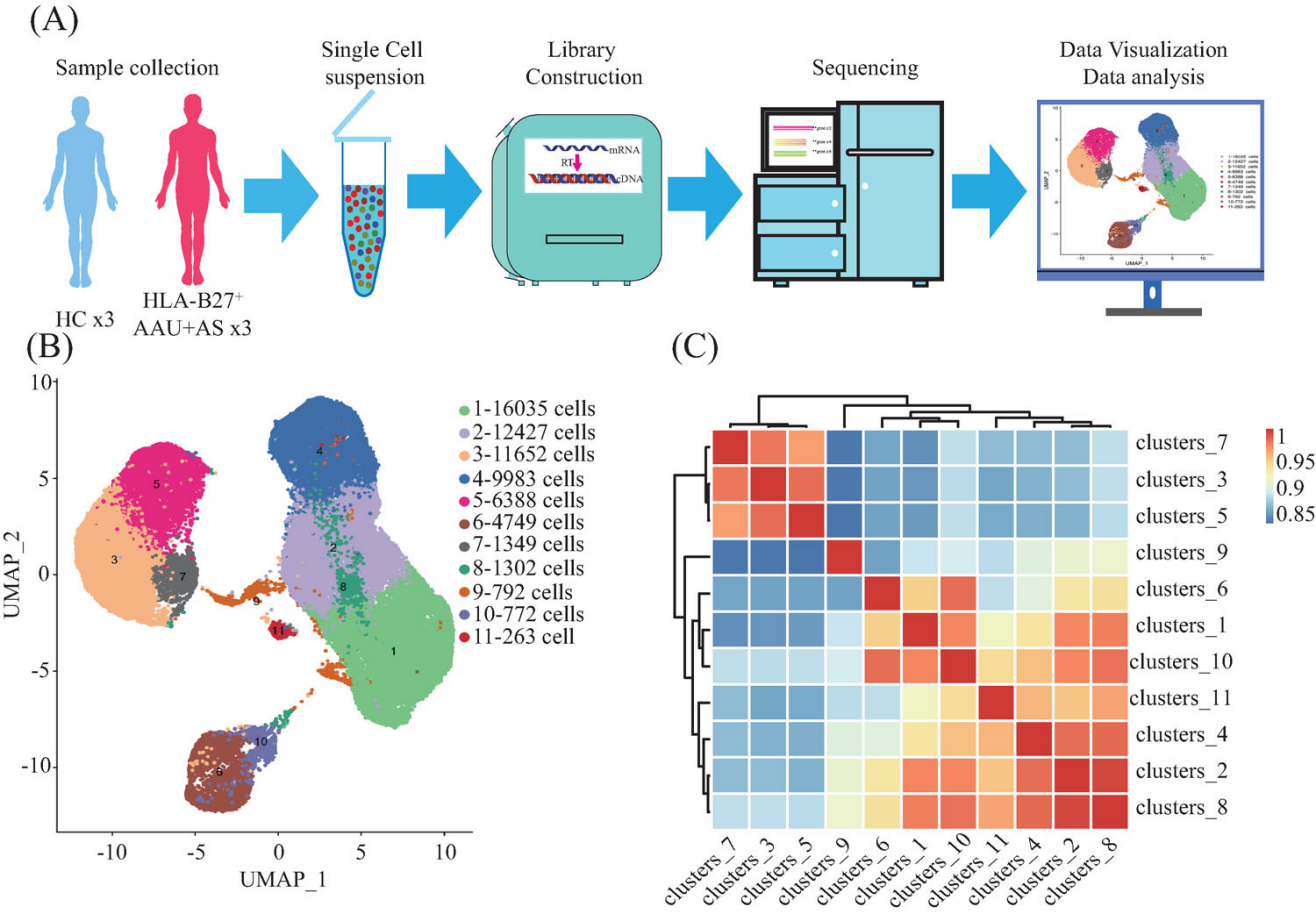
Unbiased characterization of the immune system across HL‐B27^+^ ankylosing spondylitis (AS)‐associated acute anterior uveitis (AAU) patients (AAU+AS) and healthy controls (HCs). (A) The procedure of single‐cell RNA sequencing (scRNA‐seq). (B) Uniform manifold approximation and projection (UMAP) plot of 65712 cells (34492 cells from AAU+AS patients and31220 cells from the HCs) passing quality control, color‐coded by the sample type of origin (left) and their associated cluster (right). Each point represents a cell, and each cell is grouped into one of the 11 clusters. (C) The correlation heatmap of the 11 clusters. The deeper the color, the higher the correlation in clusters. Red bands represented positive correlation, and blue bands represented negative correlation.

**FIGURE 2:**
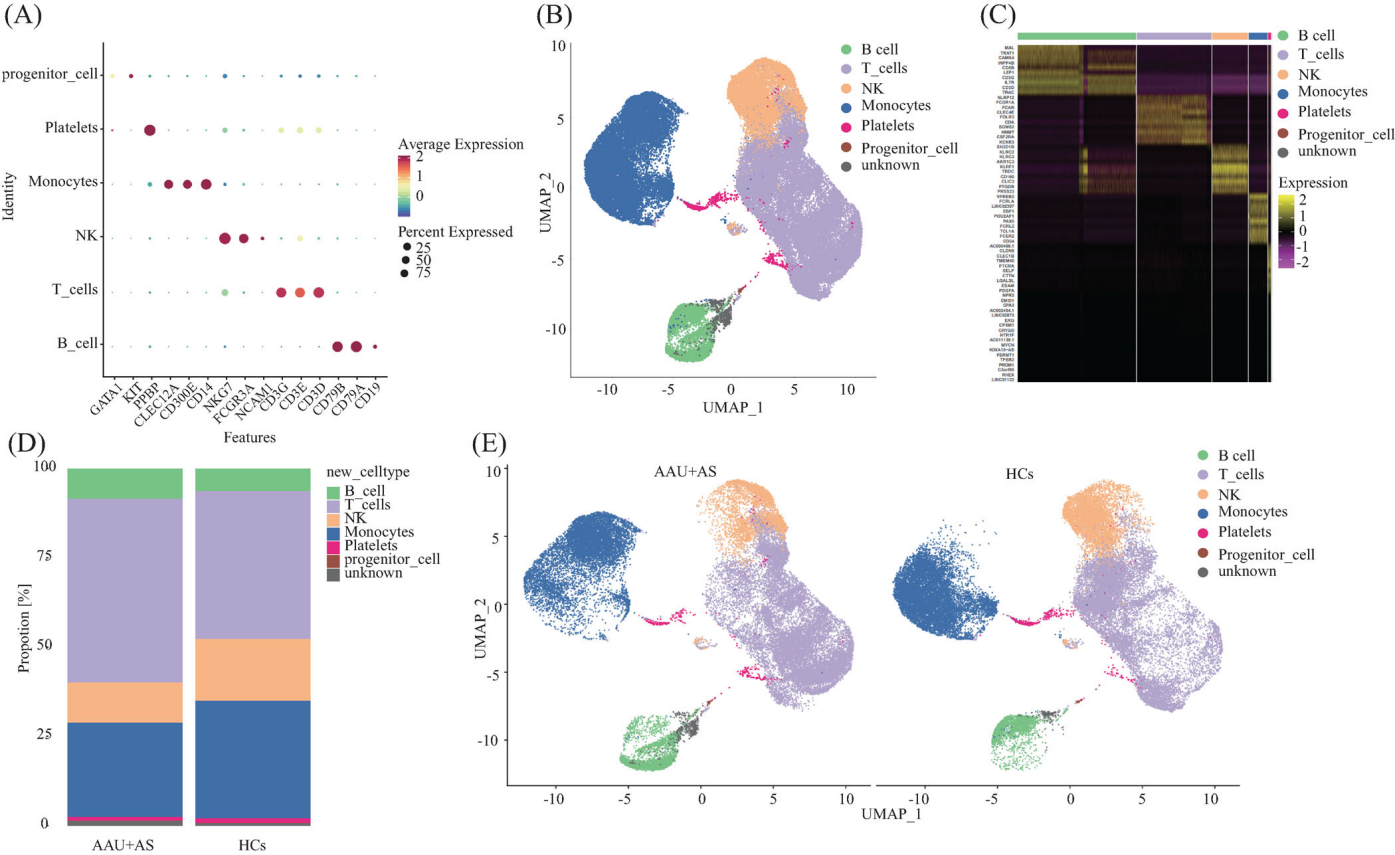
Cell types identified in peripheral blood mononuclear cells (PBMC) by UMAP. (A) Dot plots illustrate expression levels of marker genes. The distribution of the cell type‐specific marker genes in all clusters of AAU+AS patients and HCs reflect their cell identities. (B) Five major immune cell types (B cells, T cells, NK cells, monocytes and platelets) were identified by the expression of known markers for each cell type. (C) The heat map reports scaled expression of discriminative gene sets for each cluster defined in A. Up to 10 discriminators of each cluster are listed next to each cluster. The color scheme is based on *z*‐score distribution from −2 (purple) to 2 (yellow). (D) Composition of the ratios of the main cell type compositions of PBMC comparing each of the AAU+AS patients to HCs. The proportion of each cell type in each sample was calculated and then compare the proportion of each cell type between cases and controls. (E) Comparing UMAP plot of identified cell types of AAU+AS patients and HCs.

Following strict quality control and selection using several criteria, we acquired transcriptomes of 16,821, 11,357, and 13,987 cells in HLA‐B27^+^ AS‐associated AAU patients (average, 1507, 1852, and 1852 genes/cell examined) and transcriptomes of 12,508, 13,206, and 12,585 cells from HCs with means of 1764, 1786, and 1668 genes per cell detected, respectively. As revealed by t‐distributed stochastic neighbor embedding plots showing single‐cell gene expression and principal component analysis, batch effect did not exist in six samples post‐normalization. For constructing the global atlas for immune cells, the data among HLA‐B27^+^ AS‐associated AAU and HCs were merged. After expression matrix dimensionality reduction, single cells were put in 2D space with uniform manifold approximation and projection (UMAP), which identified 11 different clusters (Figure 1B). A correlation heatmap exhibiting these 11 clusters is shown in Figure 1C. In these samples, we identified five distinct immune cell types based on specific high‐expression markers on the surface of each cluster. These five cell types include T cells (clusters 1, 2, 8) identified by CD3D, CD3E, and CD3G; monocytes (clusters 3, 5, 7) identified by CD14, CD300E, and S100A12; natural killer (NK) cells (cluster 4) detected with NCAM1 and NKG7; B cells (cluster 6) discovered using CD19, CD79A, and CD79B; and platelets (cluster 9) identified by pro‐platelet basic protein (PPBP) (Table 2 and Figure 2A). Meanwhile, cluster 10 was identified as unknown cells due to its high expression of multiple cellular markers, and a small group of cells (68) was identified as progenitor cells (Figure 2B). Those 10 most significant marker genes in five cell types are shown in Figure 2C. For evaluating blood immune system state in HLA‐B27^+^ AS‐associated AAU patients, those 5 major cell types in HLA‐B27^+^ AS‐associated AAU patients were compared with those in HCs. We found an expansion of B and T cells and decreased monocytes, NK cells, and platelets in patients with HLA‐B27^+^ AS‐associated AAU (Figure 2D). Notably, significant differences in monocyte profiles were observed between patients and HCs (Figure 2E). To explore the monocyte immune features in HLA‐B27^+^ AS‐associated AAU patients, the monocytes were focused for further detailed analysis.

**TABLE 2:** Markers of each cell type.

Potential cell type	Markers
B cells	CD79A, CD79B, CD19
T cells	CD3D, CD3E, CD3G
NK cells,	NKG7, FCGR3A, NCAM1
Monocytes	CLEC12A, CD300E, CD14
Platelets	PPBP
Progenitor	GATA1, KIT

### 2.3 Mapping the monocyte atlas of HLA‐B27^+^ AS‐associated AAU patients compared with HCs

**FIGURE 3:**
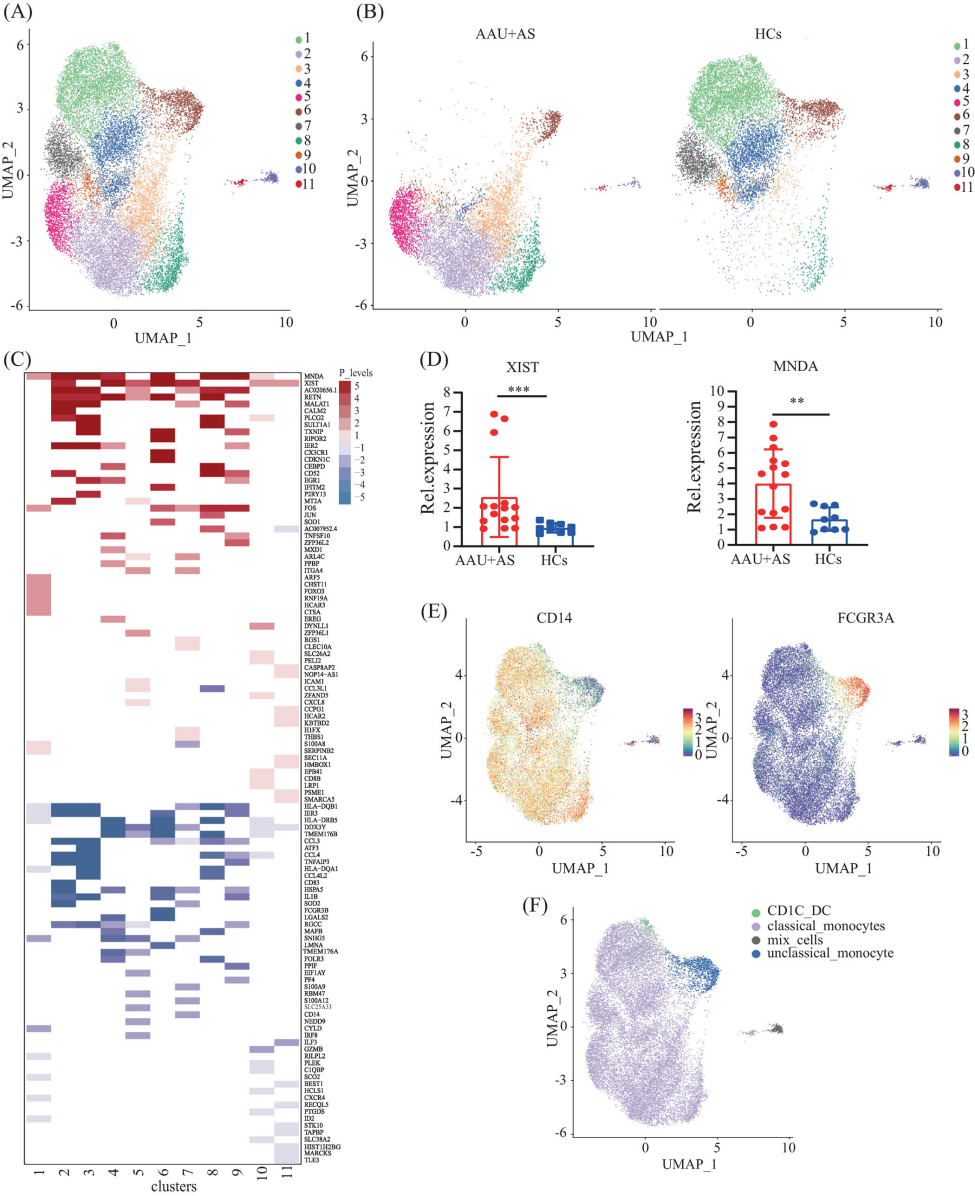
Unbiased characterization of the monocytes across AAU+AS patients and HCs. (A) UMAP plot of 19389 monocytes from AAU+AS patients and HCs. Each point represents a cell, and each cell is grouped into one of the 11 clusters. (B) Comparing UMAP plot of 11 cluster of AAU+AS patients and HCs. (C) Comparing top 50 coregulation and unique‐regulation of differentially expressed genes (DEGs) of 11 subclusters in monocytes between AAU+AS patients and HCs. Red bands indicated upregulated genes, and blue bands indicated downregulated genes in the two groups. (D) Quantitative real‐time PCR were further performed to detect the mRNA expressions of MNDA and XIST compared HL‐B27^+^ AS‐associated AAU patients with HCs. **p < 0.01, ***p < 0.001. (E) Expression pattern of two marker genes (CD14 and CD16/FCGR3A) of human monocytes. Yellow is used to represent the expression of these two marker genes, and each dot represents an individual cell. (F) The monocyte profile. Two major monocytes (classical and unclassical monocytes) were identified.

The monocytes are originally classified as two types, including CD16‐expressing and non‐CD16‐expressing. Of them, the CD16‐expressing monocytes can be further classified as two subtypes according to CD14 expression, which form the existing taxonomy including classical (CD14^++^ CD16^−^), intermediate (CD14^++^ CD16^+^), and nonclassical (CD14dim CD16^++^) monocytes.^25^ For describing differences in the HLA‐B27^+^ AS‐associated AAU patients’ monocytes at a single‐cell level, we applied UMAP algorithm in clustering cells that exhibited close expression profiles through dimensionality reduction. A total of 19,389 monocytes were further divided into 11 subclusters (Figure 3A). UMAP plots and cell cluster composition ratios for each of the six samples have been included to demonstrate the consistency within each group (Figure S1). We found significant differences in subcluster profiles between the patients and HCs (Figure 3B), indicating notable distinctions in the immune cell landscape between the two groups. In the AAU+AS group, clusters 2, 3, 5, and 8 are prominently represented, whereas the distribution of clusters in the HCs is more concentrated in clusters 1, 4, 6, and 7. In addition, trajectory analysis of subclusters reveals potential state transitions between monocyte subcluster 1 and subcluster 2, indicating dynamic changes in activation or functional states of monocytes in AAU+AS patients (Figure S2). The coregulation and unique‐regulation of DEGs in these 11 subclusters are shown in Figure 3C. Moreover, we validated the TOP coregulation DEGs in these 11 subclusters and found that the expression of X‐inactive specific transcript (XIST) and myeloid cell nuclear differentiation antigen (MNDA) genes was increased in AS‐associated AAU (AAU+AS) patients’ PBMCs (Figure 3D), potentially due to their upregulation in monocytes. We further identified two distinct monocyte subtypes based on specific high‐expression markers on the surface of these 11 clusters. Subcluster 6 was identified as CD16^hi^ monocytes, while the other subclusters were identified as CD14^hi^ monocytes, except for subcluster 10, identified as mixed cells. Moreover, a small group of cells included in subcluster 1 was identified as CD1C^+^ DCs (Figure 3E,F).

**FIGURE 4:**
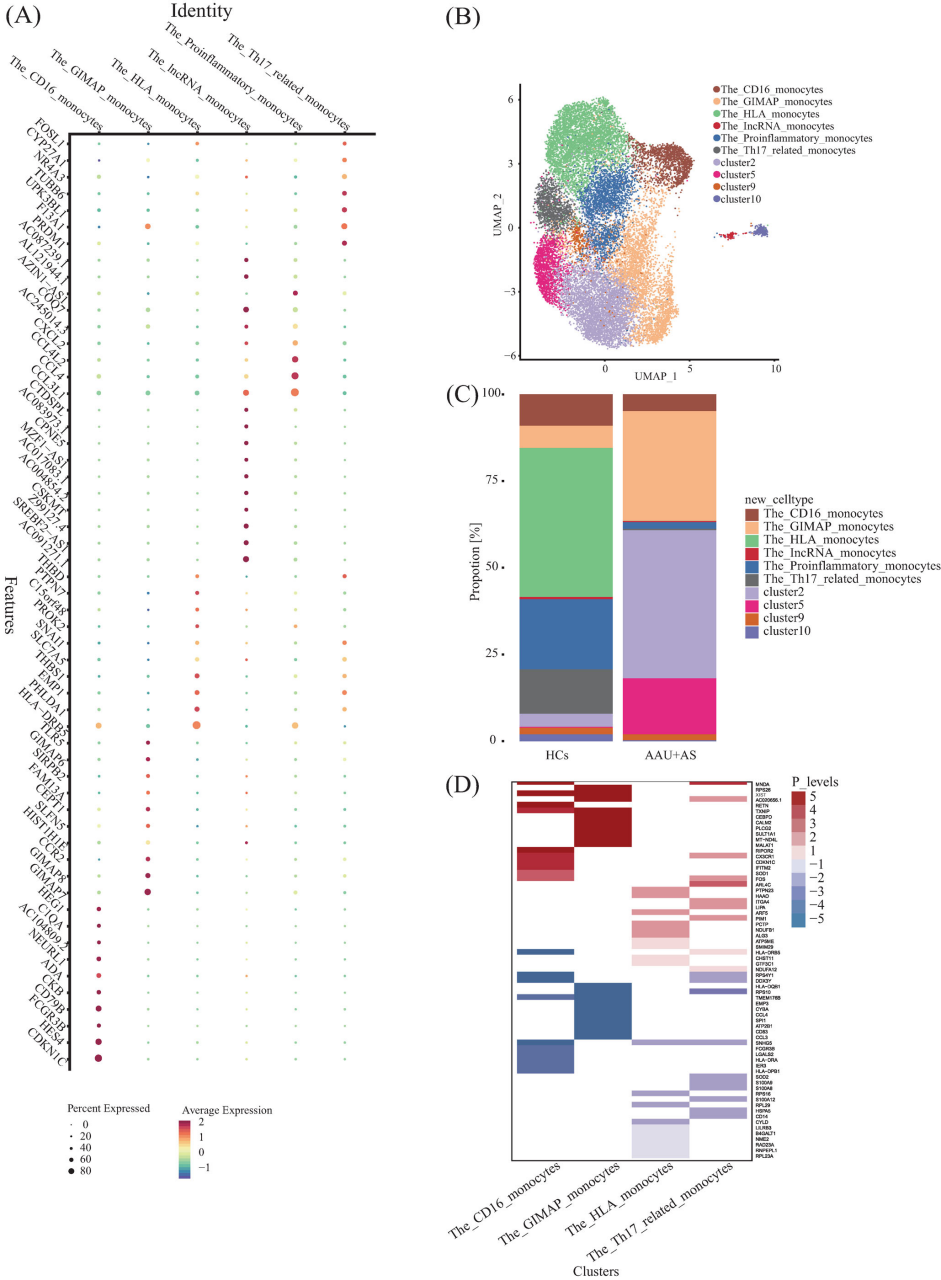
Monocyte atlas of AAU+AS patients compared with the HCs. (A) Dot plot shows the top 10 marker genes revealed by scRNA‐seq of monocyte subsets defined in B. (B) UMAP visualization of the transcriptional heterogeneity of monocytes. The 19389 monocytes are further divided into six subsets and four unidentified clusters, and their names are annotated on the right. Different colors are used to distinguish each cluster. (C) Statistical map of the identified subtypes proportion differences. (D) comparing top 25 coregulation and unique‐regulation of DEGs in HLA monocytes, GIMAP monocytes, CD16 monocyte, and Th17‐related monocytes between AAU+AS patients and HCs. Red bands indicated upregulated genes, and blue bands indicated downregulated genes in the two groups.

We further characterized diverse clusters using those 10 most significant marker genes (Table S1 and Figure 4A). According to the marker genes in each cluster, clusters with similar gene expression profiles were combined, and six main cell subtypes in the monocytes were identified. The HLA monocytes (cluster 1, except for the CD1C^+^DC) highly expressed HLA‐DRB5. Additional cell subpopulations also expressed HLA‐associated genes, but the HLA subset exhibited the highest diversity and upregulation of HLA‐associated DEGs expression in HLA‐B27^+^ AS‐associated AAU patients compared with HCs. The GTPase immune‐associated proteins (GIMAP) (clusters 3 and 8) were mainly expressed as members of the GIMAP family, including GIMAP 6, 7, and 8. GIMAP has been identified as the immune‐associated protein GTPase, which has an important effect on immune mechanisms. The proinflammatory monocytes (cluster 4) expressed a special gene combination, namely, chemokine ligands, interleukins, as well as lncRNAs (e.g., CCL3L1, CCL4, CCL4L2, CXCL2) related to virus infection,^26^ inflammation,^27^ and pyroptosis.^28^ The CD16 monocytes (cluster 6) comprised the nonclassical subtype. Apart from verifying the FCGR3B level, this work identified CDKN1C, HES4, CD79B, CKB, ADA, NEURL1, C1QA, and HEG1 as selective molecular markers. Among them, two markers, including CDKN1C and HES4, have been reported in previous studies on CD16 monocytes.^29^ The most highly expressed marker in Th17‐related monocytes (cluster 7) was PRDM1. Prdm1, a transcription factor, is an important factor driving IL‐23‐induced inflammatory activity in Th17 cells and works synergistically with RORγt for activating Th17 inflammatory process.^30^ The lncRNA monocytes (cluster 11) highly expressed diverse lncRNAs, which are less expressed in other clusters. This specific lncRNA expression pattern may be related to the unique functions of cluster 11 in immune regulation. The remaining clusters (clusters 2, 5, 9) had no distinct features, so the cell subtypes were not defined (Figure 4B). Subsequently, *t*‐test was performed to count the cell proportion of the six identified subtypes among HLA‐B27^+^ AS‐associated AAU patients relative to HCs. We found that the cell proportions of HLA monocytes (cluster 1) and CD16 monocytes (cluster 6) were significantly elevated, while the GIMAP monocytes (cluster 3) and Th17‐related monocytes (cluster 7) were significantly decreased in HLA‐B27^+^ AS‐associated AAU patients (Figure 4C).

### 2.4 Gene expression differences between HLA‐B27^+^ AS‐associated AAU and HC groups in monocytes

For obtaining more data regarding cell proportion alterations under disease conditions, DEG analysis was conducted to explore the disease processes of the defined subtypes with significant cell proportion changes: HLA monocytes (cluster 1), GIMAP monocytes (cluster 3), CD16 monocytes (cluster 6), and Th17‐related monocytes (cluster 7). We defined genes with significant upregulated and downregulated expressions in the HLA‐B27^+^ AS‐associated AAU patients compared with HCs as DEGs. Upon the fold change (FC) ≥ 2 and *p* < 0.05 thresholds, the HLA monocytes (cluster 1) had most DEG changes alterations across the subtypes, which included upregulated (245) and downregulated (183) genes. Consequently, antigen presentation probably has active participation in HLA‐B27^+^ AS‐associated AAU development. We further analyzed the top 25 DEGs, including upregulated and downregulated genes, in these identified subtypes (Table S2). Comparing the coregulation and unique‐regulation of DEGs, we found that several DEGs (CX3CR1, CDKN1C, IFITM2, SOD1, etc.) were specifically expressed in HLA monocytes, while CALM2, PLCG2, SULT1A1, MT‐ND4L, MALAT1, and others were specifically expressed in GIMAP monocytes. PTPN23, HAAO, and others were specifically expressed in CD16 monocytes. ARL4C, ITGA, LIPA, and others were specifically expressed in Th17‐related monocytes. The DEGs (MNDA, XIST, and AC020656.1) were coregulated in these four subtypes (Figure 4D).

### 2.5 DEGs in Gene Ontology and Kyoto Encyclopedia of Genes and Genomes analyses

**FIGURE 5:**
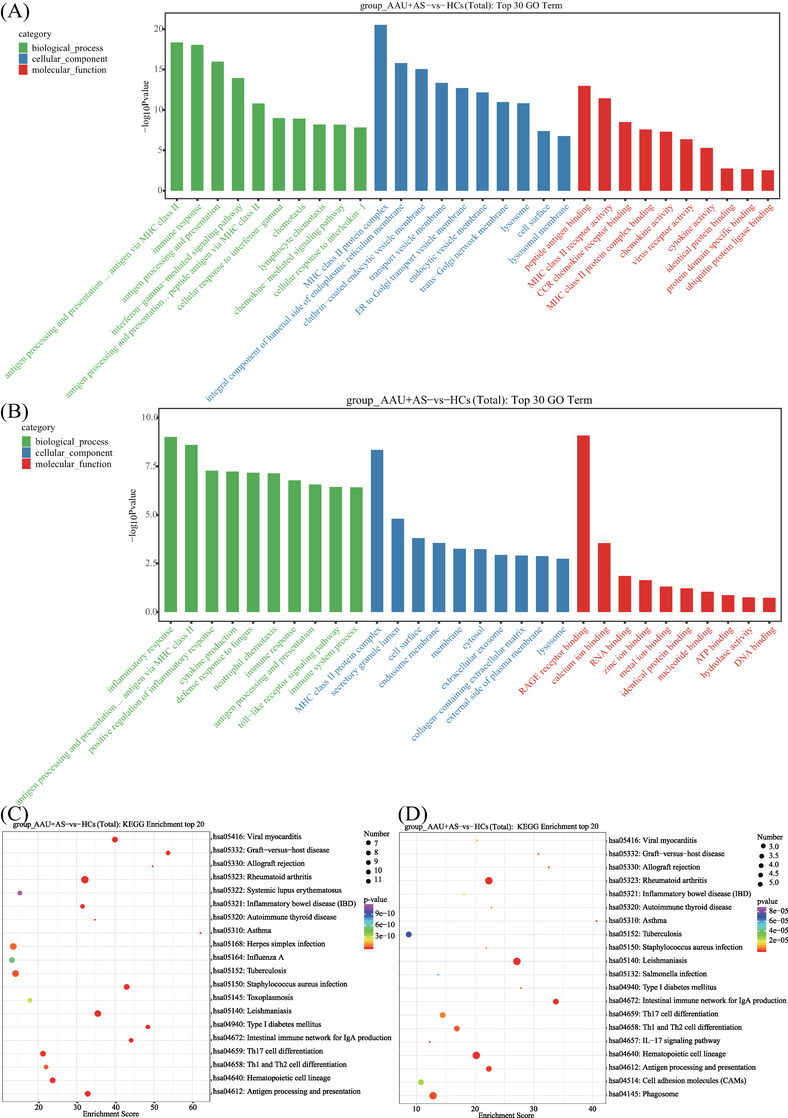
Functional analysis of AAU+AS patients compared with HCs. (A) The top 30 Gene Ontology (GO) term in HLA monocytes. Green bands indicated biological process, blue bands represented cellular component and red bands represented molecular function. The length of bands is based on −log10 *p* value from 0 to 20. (B) The top 30 GO term in GIMAP monocytes. (C)The top 20 Kyoto Encyclopedia of Genes and Genomes (KEGG) enrichment in HLA monocytes. Bubble color was based on raw *p* value. Dot size was based on hits. The deeper the color and the bigger the dots, the greater the difference in pathways. (D) The top 20 KEGG enrichment in GIMAP monocytes.

**FIGURE 6:**
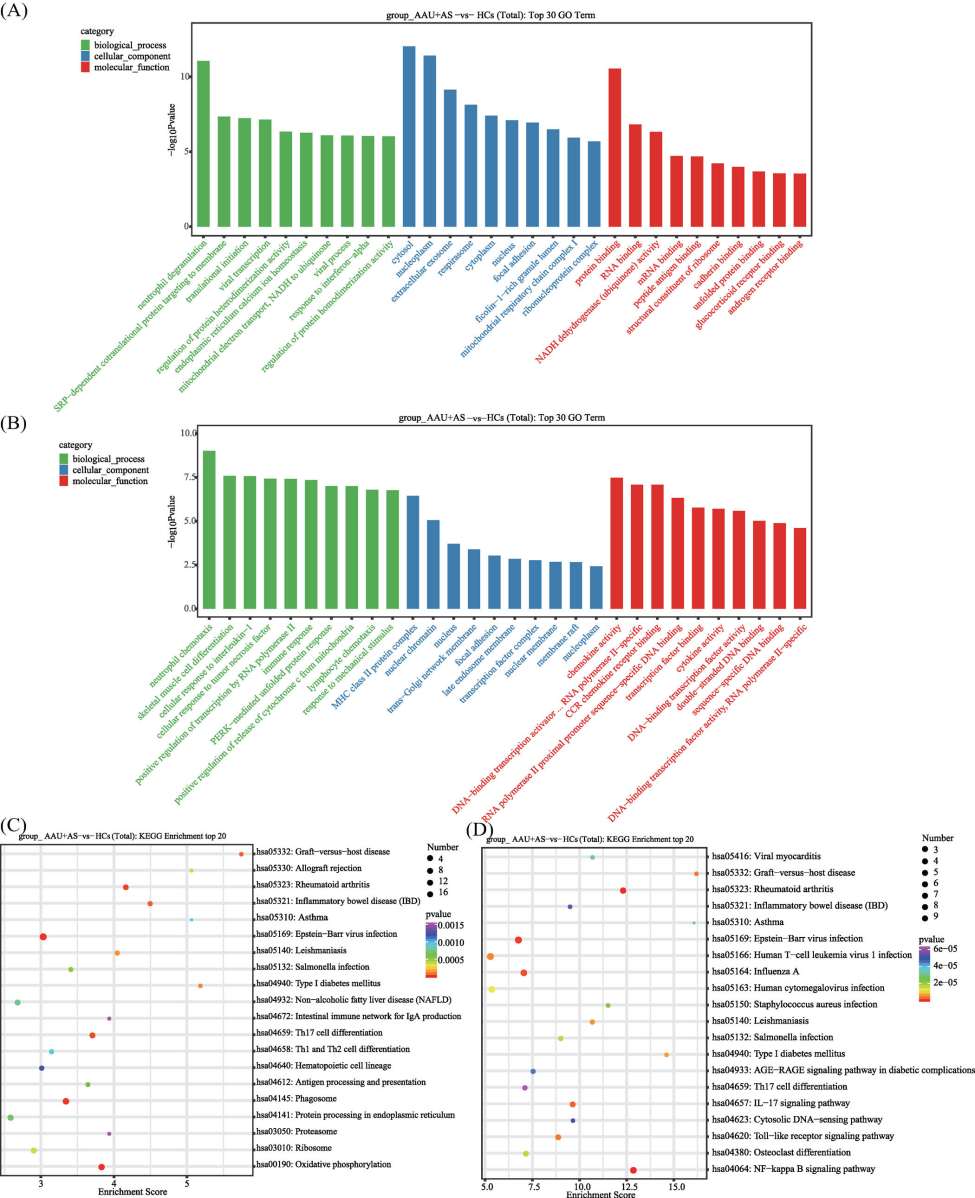
Functional analysis of AAU+AS patients compared with HCs. (A) The top 30 GO term in CD16 monocytes. Green bands indicated biological process, blue bands represented cellular component and red bands represented molecular function. The length of bands is based on −log10 *p* value from 0 to 20. (B) The top 30 GO term in Th17‐related monocytes. (C) The top 20 KEGG enrichment in CD16 monocytes. Bubble color was based on raw *p* value. Dot size was based on hits. The deeper the color and the bigger the dots, the greater the difference in pathways. (D) The top 20 KEGG enrichment in Th17‐related monocytes.

For investigating HLA‐B27^+^ AS‐associated AAU disease‐associated cell functional state and candidate molecular regulatory factors, Gene Ontology (GO) and Kyoto Encyclopedia of Genes and Genomes (KEGG) analyses were conducted on DEGs in HLA‐B27^+^ AS‐associated AAU versus HC groups. We found that those markedly associated pathways were mostly related to six pathways over‐activation: (1) MHC class II protein complex (upregulated within GIMAP monocytes, CD16 monocytes, and Th17‐related monocytes); (2) RA (highly expressed in HLA monocytes, GIMAP monocytes, CD16 monocytes, and Th17‐related monocytes); (3) Th1, Th2, and Th17 differentiation (highly expressed in HLA monocytes, CD16 monocytes, and Th17‐related monocytes); (4) antigen processing and presentation (highly expressed in HLA monocytes, CD16 monocytes, and Th17‐related monocytes); (5) Epstein–Barr virus infection (highly expressed in HLA monocytes and GIMAP monocytes); and (6) leishmaniasis (highly expressed in CD16 monocytes and Th17‐related monocytes). Moreover, most of the genes involved in these pathways were downregulated in the HLA‐B27^+^ AS‐associated AAU patients compared with the HCs (Figures 5 and 6).

## 3 DISCUSSION

HLA‐B27^+^ AS‐associated AAU represents the complicated autoimmune disorder. Dysfunction of immune cells and aberrant levels of the critical signaling factors are critical for the pathogenic mechanism of HLA‐B27^+^ AS‐associated AAU. In the current study, we revealed an insight into detailed single‐cell expression profiles of PBMCs among HLA‐B27^+^ AS‐associated AAU patients and HCs. We analyzed 65,712 cells (34,492 cells from HLA‐B27^+^ AS‐associated AAU patients and 31,220 cells from the HCs). In total, 11 clusters were identified, encompassing five main immune cell types: B cells, T cells, NK cells, monocytes, and platelets. We also found significant differences in monocyte profiles between the patients and HCs. Monocytes play a crucial role in human peripheral blood cells, essential for pathogen sensing, antigen presentation, and phagocytosis; however, functional differences across diverse subsets remains largely unclear. Remarkably, this study uncovered six monocyte subsets, including three previously unidentified subsets, and obtained special gene expression profiles. We provided further and distinct subdivisions of the current monocyte types in uveitis. Additionally, our results showed markedly altered gene expression profiles and signaling pathways in the identified monocyte subsets of HLA‐B27^+^ AS‐associated AAU patients compared with HCs.

For the CD14^high^ monocytes, including classical and intermediate subtypes, previously studies have verified their markers (S100A12, S100A8, S100A9, VCAN, LYZ, CD14, FCB1, CSTA, MS4A6A, CXCL8, CTSS, LGALS1, CST3, and MNDA)^31^, ^32^, ^33^; besides, many markers were also identified in the present work, including HLA‐ADRB5, ERG1, GIMAP6, GIMAP7, GIMAP8, CCL3L1, CCL4, and PRDM1. For CD16^high^ monocytes, the high‐throughput results verified the previously identified genes (FCGR3A, TCF7L2, MS4A7, RHOC, IFITM3, CDKN1C, HES4, SMIM25, MTSS1, BCL2A1, RRAS, and CSF1R)^34^ and obtained distinct biomarkers, including CD79B, CKB, ADA, NEURL1, C1QA, and HEG1. Villani et al.^34^ analyzed 372 single blood monocytes and identified four subtypes distinguished by 102 classifier genes in a donor through scRNA‐seq. Moreover, Hu et al. profiled 11,259 circulating monocytes and obtained six subtypes, namely, S100A12, HLA, CD16, proinflammatory, megakaryocyte‐like, and NK‐like monocytes. Moreover, they discovered another two unidentified subsets, namely, HLA and megakaryocyte‐like monocytes. They also found that proinflammatory monocytes implicate immune‐activation during Vogt–Koyanagi–Harada (VKH) disease.^29^ In the current study, we profiled 19,389 monocytes from HLA‐B27^+^ AS‐associated AAU patients and HCs and identified six subtypes, namely, HLA monocytes, CD16, proinflammatory, GIMAP, Th17‐related, and lncRNA subtypes. Compared with previous studies, we identified three new subtypes, namely, GIMAP, Th17‐related, and lncRNA subtypes. Due to differences in the disease, subtypes with different characteristics were defined. In a previous study, the GIMAP cluster has been identified as the new susceptibility locus of Behçet's disease (BD), and it is related to T‐cell survival, while T‐cell abnormality may induce BD occurrence.^35^ As is known to us, uveitis exhibits the highest prevalence among ocular symptoms of BD. NR4A3 and SLC7A5 consisted of the top 10 markers in Th17‐related monocytes. According to Owada et al.,^36^ SLC7A5 plays a significant role in Th17‐triggered autoimmune arthritis among SKG mice, the human RA animal model. Moreover, Li et al.^37^ found that miR‐106b‐5p induces the Treg/Th17 immune imbalance via NR4A3. These results indicate that Th17‐related monocytes are related to the pathogenic mechanism in HLA‐B27^+^ AS‐associated AAU by mediating the Th17 pathway. Lu and Lu^38^ reported that lncRNAs and their underlying mechanisms offer new insights into preventing and treating noninfectious uveitis among AS, BD, and sarcoidosis cases. Thus, we can infer that the three previously unexplored subtypes, namely, GIMAP, Th17‐related, and lncRNA subtypes, may play vital roles in the development of HLA‐B27^+^ AS‐associated AAU.

Many DEGs were obtained in the HLA‐B27^+^ AS‐associated AAU versus HC groups in the six identified subtypes. HLA monocytes drew our attention for exhibiting the largest number of DEGs among all the subsets. The greatest upregulated gene in the HLA monocytes is the FOS gene, with the highest FC in HLA‐B27^+^ AS‐associated AAU patients compared with HCs (FC = 3.3). In a previous study, c‐Fos was shown to be upregulated in an endotoxin‐induced uveitis (EIU) model, which was a critical approach to study human uveitis.^39^ As the calcium‐binding protein, S100A8 is mostly expressed within monocytes and granulocytes, and it exerts an essential effect on regulating immune response and inflammatory processes. In an EIU model, S100A8‐positive monocyte sand granulocytes dramatically elevated within cornea, iris‐ciliary body, and the blood.^40^ Consistent with the previous study, S100A8 was significantly increased in HLA monocytes. However, we found that Th17‐related monocytes showed markedly reduced expression levels of S100A8, S100A9, and S100A12 in HLA‐B27^+^ AS‐associated AAU patients compared with HCs. Moreover, Th17‐related monocytes that highly express lncRNA XIST, CLEC10A, CX3CR1, and THBS1, which are considered critical alarmins that induce inflammation, may be the most feasible hub genes for AS‐associated uveitis and have been found to participate in Th17 cell differentiation.^38^, ^41^, ^42^, ^43^ As revealed by the integrative analysis on biological process, Th17‐related monocytes exerted dual‐regulation on neutrophil chemotaxis, immune response, and other pathways in HLA‐B27^+^ AS‐associated AAU rather than promoting or suppressing inflammation alone. Alternatively, the downregulated genes in monocytes of HLA‐B27^+^ AS‐associated AAU patients included CCL3, CCL4, CXCL2, CXCL8, TNF, and IL1B. The above alterations conformed to two mainstream hypotheses suggesting the potential role of virus infection as an inducer for initiating uveitis and emphasizing the important effect of immune response on disease pathogenesis. Consequently, the above findings suggest that diverse monocyte subsets result in different functions in HLA‐B27^+^ AS‐associated AAU, shedding light on the precise pathogenesis of autoimmune diseases.

After the GO and KEGG annotations, DEGs in the six identified monocytes were specifically enriched in MHC class II protein complex, RA, Th1, Th2, and Th17 differentiation, Epstein–Barr virus infection, antigen processing and presentation, and leishmaniasis. Most of genes involved in these pathways were downregulated in HLA‐B27^+^ AS‐associated AAU patients compared with the HCs. Additionally, DEGs related to MHC class II exhibited 15 genes within the monocytes, including HLA‐DQB1, HLA‐B, HLA‐DRA, HLA‐DPB1, HLA‐DPA1, HLA‐DMA, HLA‐A, HLA‐DQA1, HLA‐E, HLA‐DMB, HLA‐DRB5, HLA‐B, HLA‐C, and HLA‐F. The above results suggest that susceptibility to HLA‐B27^+^ AS‐associated AAU is related to the DM, DP, DQ, and DR loci in the HLA genes. The MHC genes, important for human immune response, are identified to be genetic risk factors in some noninfectious uveitis types. Previously, chorioretinopathy, BD, and AAU have been suggested to be tightly related to alleles specific to MHC class I. Additionally, VKH disease and sarcoidosis are related to alleles specific to MHC II.^44^ The above results suggest the involvement of MHC class II molecules and HLA genes in the pathogenic mechanism of HLA‐B27^+^ AS‐associated AAU, which provide a novel direction for diagnosing and treating HLA‐B27^+^ AS‐associated AAU.

There are certain limitations in this work. Our study, which included two patients diagnosed with active HLA‐B27^+^ AS‐associated AAU and one with inactive disease, reveals that molecular and cellular changes predominantly correlate with clinically severe active disease. We acknowledge that our small sample size may limit the generalizability and statistical significance of our findings. Despite this, the diversity in disease activity stages provides essential preliminary data to understand the mechanisms operating at different phases of the disease. This underscores the need for a more balanced sample distribution in future research. Furthermore, we did not account for gender imbalance in our study. This gender‐specific sampling may introduce a bias, as sex‐based differences in immune responses are well‐documented and could potentially influence the observed molecular and cellular profiles. To enhance the reliability and applicability of our results, we plan to expand the sample size and ensure a representative distribution of disease activity states and gender in subsequent studies. In addition, while this study reveals specific molecular characteristics of HLA‐B27^+^ AS‐associated AAU, the lack of other types of uveitis patients as control groups means our conclusions should be interpreted with caution. These changes may be common in other types of uveitis as well. Therefore, future studies should include other types of uveitis patients as control groups to more comprehensively validate the specificity of these molecular characteristics. Lastly, the gene expression changes and single‐cell characteristics revealed in this study are primarily based on data from HLA‐B27^+^ AS‐associated AAU patients, without directly assessing their AS activity status. Future studies should incorporate AS activity scores and clinical data to determine whether the observed changes are directly associated with active AS. This will further aid in understanding the potential link between HLA‐B27^+^ AS‐associated AAU and active AS.

In summary, the present work fist conducted scRNA‐seq analysis on human PBMCs in HL‐B27^+^ AS‐associated AAU patients and HCs, which might lead to unbiased de novo identification of diverse cell types and states. Cell type‐specific monocyte transcriptional programs were constructed in HL‐B27^+^ AS‐associated AAU patients, which identified three subsets exhibiting special gene expression profiles uncovered previously. The identified molecular markers, such as the enhanced expression of XIST and MNDA genes in HLA‐B27^+^ AS‐associated AAU patients, can serve as diagnostic biomarkers to aid in the more accurate diagnosis of AAU. Additionally, the gene expression patterns and activation states of specific monocyte subsets can provide prognostic information, aiding in patient stratification and management. Future research will include further validation of these markers and pathways, conducting longitudinal studies, and investigating other cohorts, including HLA‐B27^+^ AS patients without AAU, to refine the specificity of the markers. Recognizing the complexity of translating molecular findings into clinical practice, we will discuss the need for large‐scale studies, the validation and standardization of diagnostic tests, and the ethical and logistical issues involved.

## 4 MATERIALS AND METHODS

### 4.1 Study population

This study was approved by the Institutional Review Board of Sichuan Provincial People's Hospital, and a total of six subjects were recruited, including three HCs and three AS‐associated AAU patients, all of whom were diagnosed as HLA‐B27 positive. The recruitment period spanned from April 2018 to July 2022. All patients provided informed consent in accordance with the ethical principles outlined in the Helsinki Declaration before analysis. Notably, none of the HLA‐B27^+^ AS‐associated AAU patients had received systemic immunosuppressive therapy, biologics, or hormones for at least 1 week prior to blood collection. Patients with malignancies or other autoimmune diseases were excluded. Additionally, exclusion criteria for the HCs included the presence of HLA‐B27^+^ AS‐associated AAU or any other autoimmune disorders. Clinical and demographic characteristics, such as age, sex, HLA‐B27 status, disease duration, leukocyte count, neutrophil count, lymphocyte count, monocyte count, ESR, CRP levels, hemoglobin, and platelets were recorded.

### 4.2 Preparation of single‐cell suspension of human PMBC samples

Fresh heparinized venous blood samples from HCs and HLA‐B27^+^ AS‐associated AAU patients were extracted into an ethylene diamine tetraacetic acid anticoagulant tube, followed by dilution of whole blood with the equivalent volume of 1×phosphate buffered saline (PBS). Afterward, lymphocyte separation solution (Ficoll) at the equivalent amount was introduced in the 50‐mL centrifuge tube, followed by slow spreading of diluted blood onto lymphocyte separation solution and 20‐min centrifugation at 2000 rpm on the horizontal rotor under 20°C. We set the break as 0. PBMCs were then carefully drawn into a new 15‐mL centrifuge tube. While 1×PBS (10 mL) was added to rinse white membrane cells, followed by another 10‐min centrifugation at 300×*g*. After discarding supernatants, cells were resuspended with 5 mL 1×PBS was added to resuspend cells, followed by another 10‐min centrifugation at 300×*g*. After washing twice, supernatants were removed, then 1 mL RPMI‐1640 medium that contained 0.04% BSA was added to resuspend cells. Single‐cell suspension concentration was determined with the luna cell counter, whereas cell viability was analyzed with the Trypan Blue stain.

### 4.3 Transcriptome amplification, library construction, and sequencing

In line with 10× Genomics Chromium Next GEM Single Cell 3′ Reagent Kits v3.1 (No. 1000268) Operation Manual for computer and library construction, we adjusted the freshly prepared single‐cell suspension at 700−1200 cells/µL. After library construction, the Illumina Nova 6000 PE150 platform was employed for sequencing. Later, cDNA was prepared from cellular mRNA by using reverse transcriptase. The 10× Genomics Single‐Cell 3′ Library V2 Kit was applied in synthesizing cDNA and constructing the library. Thereafter, the BGISEQ‐500 sequencer was used to sequence both cDNA libraries as 100‐bp paired‐end reads (Figure 1A).

### 4.4 Bioinformation analysis process

#### 4.4.1 Processing of scRNA‐seq data

OE Biotech Co., Ltd. (Shanghai, China) was responsible for sequencing the database and analyzing data. We obtained original reads obtained through high‐throughput sequencing in a fastq format. Cell Ranger (v5.0.0), the official software of 10× genomics, was adopted for analyzing raw data quality and comparing data from reference genome. The software identifies the barcode marker distinguishing cells within the sequence and unique molecular identifiers (UMI) markers for diverse mRNA molecules in every cell to quantify high‐throughput single‐cell transcriptome data, so as to obtain quality control data like sequencing saturation, gene median value, and high‐quality cell count.

#### 4.4.2 Gene quantitative quality control and data preprocessing

In line with cell ranger preliminary quality control, we utilized Seurat (v4.0.0) software to further process data for quality control. In theory, the number of genes expressed by most cells, the UMI number, and the mitochondrial transcript proportion are concentrated within one specific region. Therefore, low‐quality cells were eliminated based on nUMI, nGene, percent, mito distribution, and other indicators. High‐quality cells were those containing > 200 retained genes, >1000 UMI, >0.7 log10GenesPerUMI, <5% red blood cell genes and <5% mitochondrial UMI. Double cells were simultaneously removed using the Doublet Finder (v2.0.2) software.

#### 4.4.3 Dimension reduction and clustering

Highly variable genes were selected using Find Variable Genes of Seurat package, and their expression patterns were utilized for mutual nearest neighbors dimensionality reduction analysis. Following dimensionality reduction, we applied single‐cell clustering algorithms to the reduced dataset to categorize cells into distinct clusters based on their gene expression profiles. This clustering step enabled us to discern heterogeneous cell populations and infer their potential biological functions. To visualize the clustering results and the high‐dimensional dataset in a more interpretable form, UMAP, a nonlinear dimensionality reduction technique, was employed. The UMAP analysis facilitated the visualization of the data in 2D space, allowing for an intuitive understanding of the cellular landscape and the spatial relationships between the identified clusters.

#### 4.4.4 Identification of marker genes

Marker genes were identified using Find All Markers function in Seurat package, in other words, genes significantly upregulated within every cell population versus others were discovered. They were the possible marker genes in every cell population. Feature Plot and Violin Plot functions were employed for visualization of marker genes.

#### 4.4.5 Cell type identification

Single R (v1.4.1) package was utilized in calculating association of cell expression profiles with reference dataset based on the single‐cell reference expression quantitative public dataset. We then assigned the most significantly associated cell type from reference dataset to cells for identification, which partially eliminated the human subjective factors‐induced interference. The principle of identification was to determine Spearman correlation of expression pattern in every cell of the sample with that from reference dataset and choose the cell type most significantly associated with expression of sample cells in dataset as the eventual cell type for identification.

#### 4.4.6 Real‐time reverse transcriptase‐polymerase chain reaction

Real‐time reverse transcriptase‐polymerase chain reaction (RT‐PCR) was carried out with the Rotor‐Gene Q (Qiagen, Germantown, MD, USA). The amplification reaction mix included the Master MixQiagen (10 µL) (Trans Gen Biotech SYBR Green Real‐Time PCR Kit, Beijing) and cDNA (1 µL,100 ng). Forty‐five amplification cycles were carried out for each sample. Results were analyzed with the 2^−ΔΔCt^ method. Quantitative PCR experiments followed the MIQE guidelines. Gene expression levels were normalized with levels of housekeeping gene (18S). Primers were purchased from Sangon Biotech. Forward and reverse primer sequence and catalogue numbers are herein listed: MNDA (Forward: AACTGACATCGGAAGCAAGAG; Reverse: CCTGATTCGGAGTAAACGAAGTG), XIST (Forward: CTACTAGCTCCTCGGACAGC; Reverse: TGTTTGCAGTCCTCAGGTCT).

#### 4.4.7 Differential gene and enrichment analysis

DEGs were screened using Find Markers function of Seurat. Significant DEGs were selected upon conditions of a *p*‐value < 0.05 and a difference multiple > 1.5 times. GO as well as KEGG enrichment of significant DEGs was conducted using hypergeometric distribution test.

### 4.5 Statistical analysis

Unpaired *t*‐tests or Mann–Whitney *U*‐tests were adopted for assessing statistical significance if necessary. SPSS 20 was employed for statistical analysis. *p* < 0.05 (two‐sided) stood for statistical significance. *p < 0.05; **p < 0.01; ***p < 0.001; ****p < 0.0001; not significant (ns) *p* > 0.05.

## AUTHOR CONTRIBUTIONS

Huan Li, Jialing Xiao, Weijia Wu, Xueming Ju, Chengzi Gan, Liang Wang, and Lixin Zhang collected the samples. Bo Gong, Ning Xiao, Bolin Deng, Liang Wang, Lixin Zhang, Xiangmei Li, Yutong Wei, Ting Wang, and Siyu Zhu were responsible for data analysis. Huan Li was in charge of manuscript writing. Bo Gong, Yu Zhou, Bolin Deng, Jing Zhu, and Xueming Ju contributed to scientific content analysis and paper revision. All authors have read and approved the final manuscript.

## CONFLICT OF INTEREST STATEMENT

The authors declare no conflict of interest.

## ETHICS STATEMENT

The human ethics committee was from the Sichuan Provincial People's Hospital Affiliated to University of Electronic Science and Technology of China (AF‐17/01.0). All protocols followed Declaration of Helsinki. Informed consent was obtained from each participant.

## Supporting information



Supporting Information

## Data Availability

All data supporting the findings of this study are available from the authors upon reasonable request. The scRNA‐seq data are openly available in the GSA‐Human (HRA008459).
